# Calcium-silicate mesoporous nanoparticles loaded with chlorhexidine for both anti- *Enterococcus faecalis* and mineralization properties

**DOI:** 10.1186/s12951-016-0224-7

**Published:** 2016-10-21

**Authors:** Wei Fan, Yanyun Li, Qing Sun, Tengjiao Ma, Bing Fan

**Affiliations:** The State Key Laboratory Breeding Base of Basic Science of Stomatology (Hubei-MOST) and Key Laboratory of Oral Biomedicine Ministry of Education, School and Hospital of Stomatology, Wuhan University, 237 Luoyu Road, Wuhan, 430079 People’s Republic of China

**Keywords:** Mesoporous, Nanoparticle, *Enterococcus faecalis*, Chlorhexidine, Mineralization

## Abstract

**Background:**

In infected periapical tissues, *Enterococcus faecalis* is one of the most common dominant bacteria. Chlorhexidine has been proved to show strong antibacterial ability against *E. faecalis* but is ineffective in promoting mineralization for tissues around root apex. Mesoporous calcium-silicate nanoparticles are newly synthesized biomaterials with excellent ability to promote mineralization and carry-release bioactive molecules in a controlled manner. In this study, mesoporous calcium-silicate nanoparticles were functionalized with chlorhexidine and their releasing profile, antibacterial ability, effect on cell proliferation and in vitro mineralization property were evaluated.

**Results:**

The chlorhexidine was successfully incorporated into mesoporous calcium-silicate nanoparticles by a mixing-coupling method. The new material could release chlorhexidine as well as Ca^2+^ and SiO_3_^2−^ in a sustained manner with an alkaline pH value under different conditions. The antimicrobial ability against planktonic *E. faecalis* was dramatically improved after chlorhexidine incorporation. The nanoparticles with chlorhexidine showed no negative effect on cell proliferation with low concentrations. On dentin slices, the new synthesized material demonstrated a similar inhibitory effect on *E. faecalis* as the chlorhexidine. After being immersed in SBF for 9 days, numerous apatite crystals could be observed on surfaces of the material tablets.

**Conclusions:**

Mesoporous calcium-silicate nanoparticles loaded with chlorhexidine exhibited release of ions and chlorhexidine, low cytotoxicity, excellent antibacterial ability and in vitro mineralization. This material could be developed into a new effective intra-canal medication in dentistry or a new bone defect filling material for infected bone defects.

## Background

*Enterococcus faecalis* (*E. faecalis*) is a gram-positive facultative anaerobe that is resistant to many commonly used antibiotics and can cause several lethal infections in human body, such as in gastrointestinal or urinary tract [1]. Similarly in dentistry, *E. faecalis* is reported to be a main cause for refractory root canal infection, and takes great responsibility for a large portion of clinical treatment failures [2, 3]. Therefore, looking for effective and efficient methods to eliminate *E. faecalis* infection inside the root canal or around the root apex has long since been an intriguing research topic for both dental and material scientists [4–7]. Despite this, the present most often used medication inside the root canal, i.e. calcium hydroxide [Ca(OH)_2_], shows slow and weak anti-bacterial ability against *E. faecalis* [8, 9]. What’s more, as the intra-canal medication is normally removed by irrigation before the canal is sealed, the substantivity on dentin surface is deemed as another important feature of intra-canal medications.

Chlorhexidine (CHX) is an effective bactericidal cationic polybiguanide showing strong anti-bacterial ability against many infectious microbes, including the *E. faecalis* [10]. The solution or gel of CHX has been used to irrigate or medicate root canals to help eliminate *E. faecalis* [11, 12]. The antimicrobial ability of chlorhexidine has been proved to be dose-dependent [13]. Besides, the CHX shows excellent substantivity on dentin surface to elongate its anti-bacterial effects for up to several weeks even after being removed [14–17]. However, CHX is always cytotoxic at relatively high concentrations and has an acidic pH, which is non-suitable for direct contact with tissues around root apex and unable to neutralize endotoxin or other inflammatory metabolic products of bacteria [18, 19]. Besides, chlorhexidine may not be suggested to be used in canal as a sole bactericidal agent for its lower efficacy compared with some other medicaments [20, 21]. As the infection in root canal often involves the peri-apical alveolar bone tissues, it is thought to be an ideal property for intra-canal medications to promote the healing of peri-apical alveolar bone. Unfortunately, CHX is of no pro-osteogenic property for infected bone tissues [22].

Calcium (Ca) and silicate (Si) ions have been proven to be effective in promoting osteogenesis both in vitro and in vivo [23, 24]. Ca–Si mesoporous nanoparticles (MCSNs) are newly synthesized materials for its continuous Ca–Si ions release and strong ability to carry-and-release various bioactive molecules in a sustained manner [25–27]. These properties enable the Ca–Si mesoporous nanoparticles to be a promising filling material candidate for bone defects. Despite this, the weakness of this material for being used in infected areas is its low anti-bacterial ability.

To overcome the weaknesses of both CHX and Ca–Si mesoporous materials, a combined strategy was designed in this study to synthesize new Ca–Si mesoporous nanoparticles loaded with chlorhexidine (M-CHX), and their antibacterial effect against *E. faecalis* and in vitro mineralization properties were investigated.

## Methods

### Synthesis and characterization of MCSNs and M-CHX

The mesoporous calcium-silicate nanoparticles were synthesized according to our previous reported publications [25, 26]. Briefly, 6.6 g cetyltrimethylammonium bromide (CTAB) (Sigma-Aldrich, St. Louis, MO, USA) and 12 mL ammonium hydroxide (Sinopharm Chemical Reagent Co. Ltd., Shanghai, China) were added to 600 mL of double-distilled water to dissolve. After stirring for 30 min, 30 mL tetraethyl orthosilicate (TEOS) (Aladdin Industrial Corporation, Shanghai, China) and 31.21 g calcium nitrate tetrahydrate (Sinopharm Chemical Reagent Co. Ltd., Shanghai, China) were added with vigorous stirring for another 3 h. The products were collected by filtration and washed three times each with double-distilled water and ethanol. Finally, the powders were dried at 60 °C overnight and calcined at 550 °C for 2 h.

The chlorhexidine digluconate (CHX) (Adamas, Basel, Switzerland) was incorporated into MCSNs at a ratio of 1:1 by mass. Briefly, MCSNs were mixed with 1 % (w/v) chlorhexidine aqueous solution with vigorous stirring for 6 h at 80 °C. After filtration, the powders were washed with ethanol for three times. Then the products were dried at 60 °C for 6 h and collected, referred to as M-CHX.

The MCSNs and M-CHX were characterized by field –emission scanning electron microscopy (FE-SEM) (Sigma, Zeiss, Germany), transmission electron microscopy (TEM) (JEM-2100, JEOL, Tokyo, Japan), and energy dispersive spectrometry (EDS) (X-Max50, Oxford Instruments, Abingdon, UK). The Brunauer–Emmett–Teller and the Barrett–Joyner–Halenda analyses were used to examine the specific surface area, pore volume, and pore size distribution by nitrogen adsorption–desorption isotherms (ASAP 2020, Micromeritics, Norcross, GA, USA). The combination between MCSNs and CHX was evaluated by Fourier transformed infrared spectroscopy (FTIR) (Nicolet5700, Thermo Fisher Scientific, Waltham, MA, USA).

### pH measurement and ion release profile

The pH change was measured by soaking 100 mg MCSNs or M-CHX in 20 mL double-distilled water, simulated body fluid (SBF), α-MEM [supplemented with 10 % fetal calf serum (FCS) (Thermo Scientific) and 1 % penicillin/streptomycin (Thermo Scientific)] and brain heart infusion (BHI) respectively at 37 °C. At 1, 3, 6, 9 days, pH measurement was performed using a pH meter (Sartorius AG, Goettingen, Germany).

For detecting the release of Ca^2+^ and SiO_3_^2−^, 20 mg MCSNs and M-CHX were soaked in 10 mL Tris–HCL (1M, pH 7.4), SBF or α-MEM at 37 °C for 1, 3, 6, 9 days. At each time point, 5 mL of the solution was retrieved for measuring the ion concentration by inductively coupled plasma optical emission spectrometry (ICP-OES) (Optima4300DV, PerkinElmer, Waltham, MA, USA) and 5 mL fresh solution was added. The total amount of ions release was calculated.

CHX release profile in different solutions was evaluated using a spectrometer (UV-2401PC, SHIMADZU CORPORATION, Japan). 20 mg M-CHX was immersed in 10 mL Tris–HCl, SBF or α-MEM at 37 °C for 1, 2, 4, 8, 12 h or 1, 3, 6, 9 days. The concentration of CHX in solution (mg/mL) was analyzed by measuring the absorbance at 255 nm. The tests were performed in triplicates.

### Antimicrobial effects against planktonic *E. faecalis*

A colony-forming units (CFUs) counting method was applied to investigate the antibacterial activity of M-CHX. A 1 mL suspension (1 × 10^4^ CFUs/mL) of *E. faecalis* (ATCC 29212, ATCC, Manassas, VA, USA) was incubated with 1, 2, 5 mg MCSNs or M-CHX for 24 h at 4 °C. Final concentrations of 0.005, 0.01 and 0.02 % CHX which were close to the possible CHX concentration released by M-CHX in BHI condition were used as controls for comparison. Subsequently, 10 μL of the solution was inoculated on a BHI agar plate and incubated for another 24 h at 37 °C anaerobically. Finally, CFUs of *E. faecalis* were counted. For each group, the test was repeated for six times.

### Cytotoxicity test

To assess the cytotoxicity of M-CHX, the cell count-ing kit-8 (CCK-8) (Dojindo Laboratories, Kumamoto, Japan) method was performed on MC3T3-E1 cells (ATCC). The extracts of nanoparticles were prepared firstly. 10 mg MCSNs or M-CHX was soaked in 1 mL α-MEM at 37 °C for 24 h. After centrifugation, the supernatant was sterilized using a 0.22 μm filter unit (Merck Millipore Ltd., Darmstadt, Germany) and supplemented with 10 % FCS. Dilutions of 2, 4, 10 mg/mL were used. For cell culture, 7 × 10^3^ cells suspended in 100 μL α-MEM with 10 % FCS and 1 % penicillin/streptomycin were seeded into each well of a 96-well plate. Six repeated wells were included for each group. After incubation at 37 °C with 5 % CO_2_ for 48 h, 100 μL fresh α-MEM and 100 μL extracts were added. Final concentrations of 0.01, 0.02 and 0.03 % CHX which were close to the possible CHX concentration released by M-CHX inα-MEM condition were tested simultaneously. After incubation for 2, 4, 6 days, cells were washed with fresh α-MEM and cultured with 100 μL α-MEM and 10 μL CCK-8 at 37 °C for 4 h. Then the absorbance at 450 nm was measured by a micro-plate reader (Power Wave XS2, BioTek Instruments, VT, USA).

### Minimum inhibitory concentration (MIC) and bactericidal concentration (MBC) of MCSNs and M-CHX extracts

MIC and MBC of MCSNs and M-CHX extracts were determined by a serial microdilution assay [28, 29]. Final concentration of 100 mg/mL nanoparticles extracts were prepared (in BHI at 37 °C for 24 h). From these starting solutions, two fold dilutions were made. Then 50 μL suspension (1 × 10^5^ CFUs/mL) of *E. faecalis* was added into each well of a 96-well plate and incubated with 50 μL dilution at 37 °C for 24 h anaerobically. An optical density measurement was conducted by a micro-plate reader at 600 nm. Wells with the lowest concentrations where no turbidity could be detected compared with controls were determined to have MIC. MBC was determined by inoculating solutions from wells without turbidity on BHI agar plates. The test was conducted for three times.

### Inhibition of *E. faecalis* on dentin

Dentin slices with a size of 4 mm (width) × 4 mm(length) × 1 mm (thickness) were prepared from extracted wisdom teeth under the approval of ethics committee of School and Hospital of Stomatology, Wuhan University. All dentin slices were cleaned by ultrasonic wash for 4 min in double-distilled water, 5.25 % sodium chloride (NaCIO), 17 % ethylenediaminetetraacetic acid (EDTA) successively and ended in double-distilled water for one more minutes. Then 100 mg MCSNs or M-CHX was mixed with 400 μL distilled water to form paste for the medication of dentin slices. For CHX group, Methyl cellulose (Aladdin Industrial Corporation, Shanghai, China) was added to 2 % CHX solution to form gel. Dentin slices embedded in paste or gel were incubated at 37 °C for 7 days. Each group included 6 slices. After being flushed by 6 mL distilled water, dentin slices were immersed in 1 mL suspension (1 × 10^4^ CFUs) of *E. faecalis* for bacterial colonization at 37 °C for 7 days. Subsequently, dentin slices were washed with 1 mL phosphate buffer saline (PBS) for three times to remove floating bacteria and added to 5 mL fresh BHI solution. At 2, 4, 6, 8 h, 1 mL suspension was taken out for an OD value measurement at 600 nm using a spectrometer. Besides, FE-SEM was conducted on one more slice in each group after bacteria incubation to check the dentin surface.

### In vitro mineralization

For the test of in vitro mineralization ability, 20 mg M-CHX or MCSNs was compacted to a tablet with a diameter of 6 mm and thickness of 0.7 mm. The tablets were soaked in SBF for 9 days at 37 °C. FE-SEM and EDS analysis were performed to check the surface of the tablet for the formation of any mineral crystals.

### Statistical analysis

The statistical analysis was performed using one-way ANOVA with a post hoc SNK test. Statistical significance was set at p < 0.05.

## Results

### Characterization of MCSNs and M-CHX

**Fig. 1:**
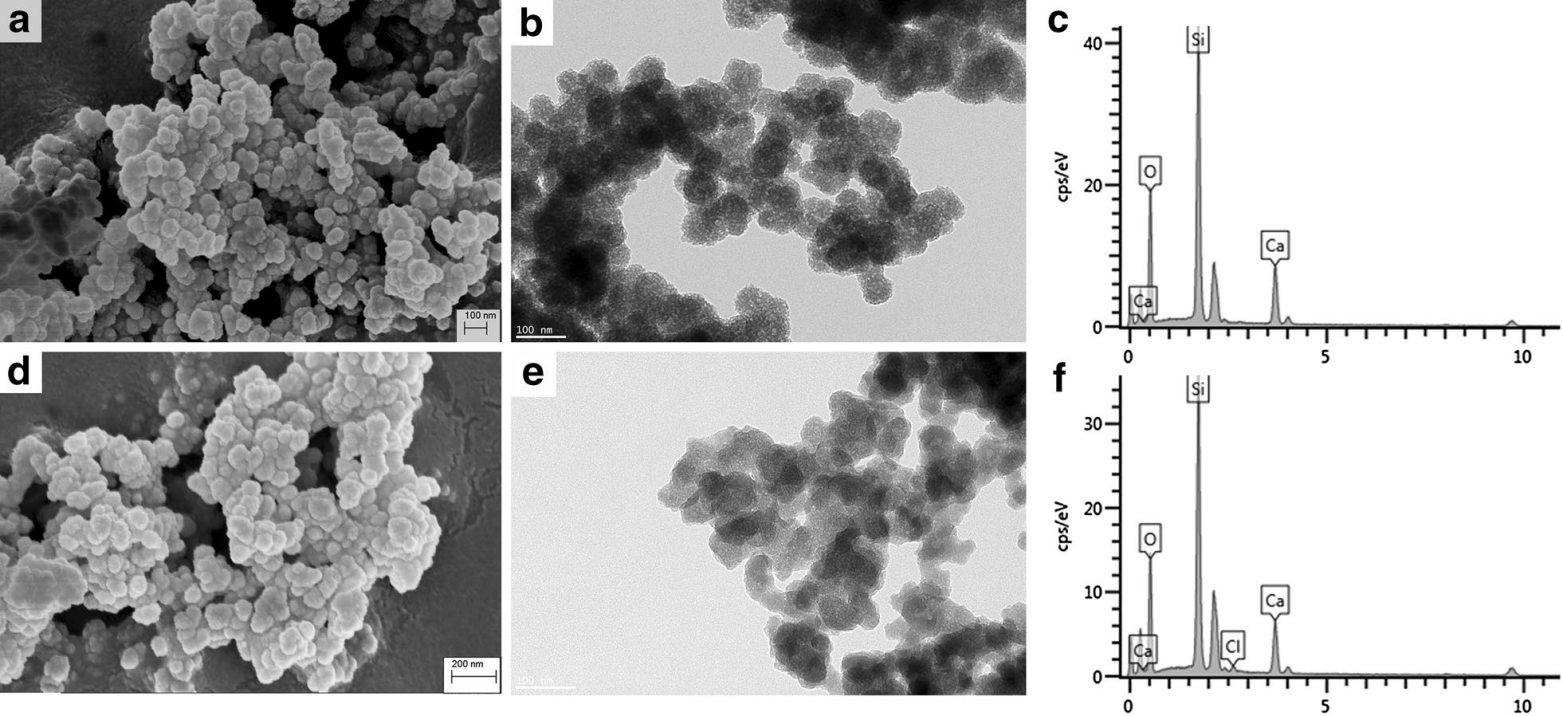
Characterization of MCSNs and M-CHX. **a** SEM image of MCSNs; **b** TEM image of MCSNs; **c** EDS analysis of MCSNs; **d** SEM image of M-CHX; **e** TEM image of M-CHX; **f** EDS analysis of M-CHX

**Fig. 2:**
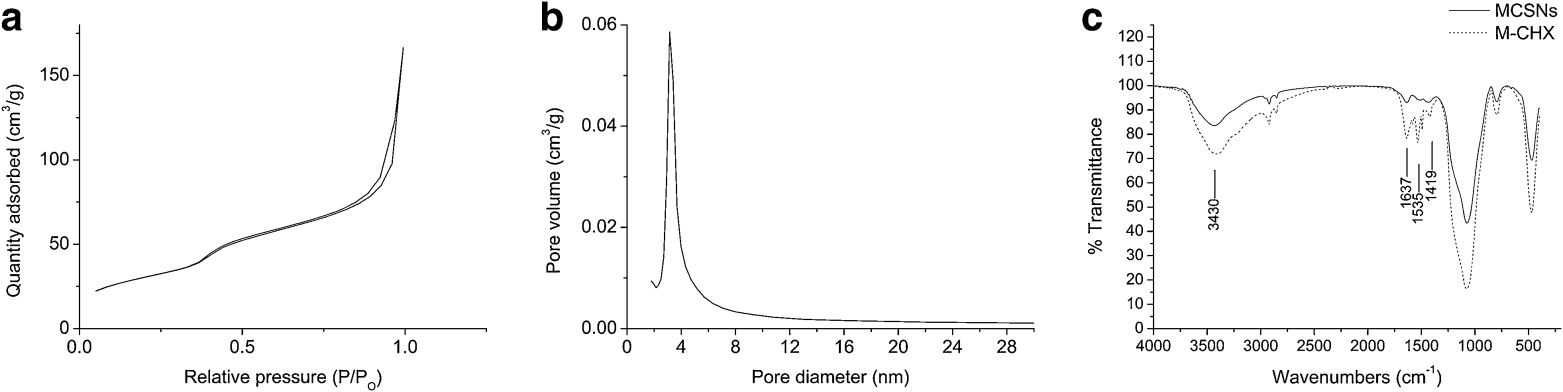
Nitrogen adsorption–desorption result of MCSNs and FTIR result. **a** Nitrogen adsorption–desorption isotherms test of MCSNs; **b** Pore size distribution of MCSNs; **c** FTIR spectrum of MCSNs and M-CHX

SEM images showed that the MCSNs and M-CHX were both in a spherical shape but strongly aggregated (Fig. 1a, d). For the statistical estimation of diameters, 150 nanoparticles in three SEM images were chosen and measured using ImageJ software. The diameter of MCSNs and M-CHX is respectively 79.5 ± 13.3 and 78.6 ± 11.1 nm. TEM images revealed mesoporous structures evenly distributed inside the MCSNs (Fig. 1b), and nitrogen adsorption–desorption isotherms indicated type isotherms with H1-type hysteresis loops (Fig. 2a). The diameter of mesoporous fell mainly in the range of 2–6 nm (Fig. 2b). The pore volume, surface area and mean pore size were shown in Table 1. TEM images of M-CHX showed that mesoporous structures were not so clear as MCSNs (Fig. 1e). EDS analysis revealed the existence of Ca and Si elements in MCSNs and Cl element in M-CHX (Fig. 1c, f). The percentage of Ca, Si, O and Cl in materials was presented in Table 2. The FTIR spectrum indicated that both MCSNs and M-CHX had an absorption peak at 3430 cm^−1^, which was the specific adsorption peak of hydroxyl groups (–OH). Absorption peaks at 1535 and 1637 cm^−1^ of M-CHX were the carbon–carbon double bonds (C=C) specific in benzene rings of chlorhexidine digluconate molecule while absorption peak at 1419 cm^−1^ was caused by the deformation vibration of –OH. Besides, absorption peaks at 1637 and 1535 cm^−1^ are also possible to be the imino groups (-NH-) existing in CHX in a large number (Fig. 2c).

**Table 1:** The surface area (S_BET_), pore volume (V_P_), and mean pore size (D_P_) of the MCSNs

	S_BET_ (m^2^ g^−1^)	V_P_ (cm^3^ g^−1^)	D_P_ (nm)
MCSNs	108.72	0.26	9.48

**Table 2:** Quantitative EDS analysis of the element proportion in MCSNs and M-CHX (weight %)

	O	Si	Ca	Cl
MCSNs	61.08	28.43	10.49	–
M-CHX	59.21	29.94	9.97	0.89

### pH and ion release profile

**Fig. 3:**
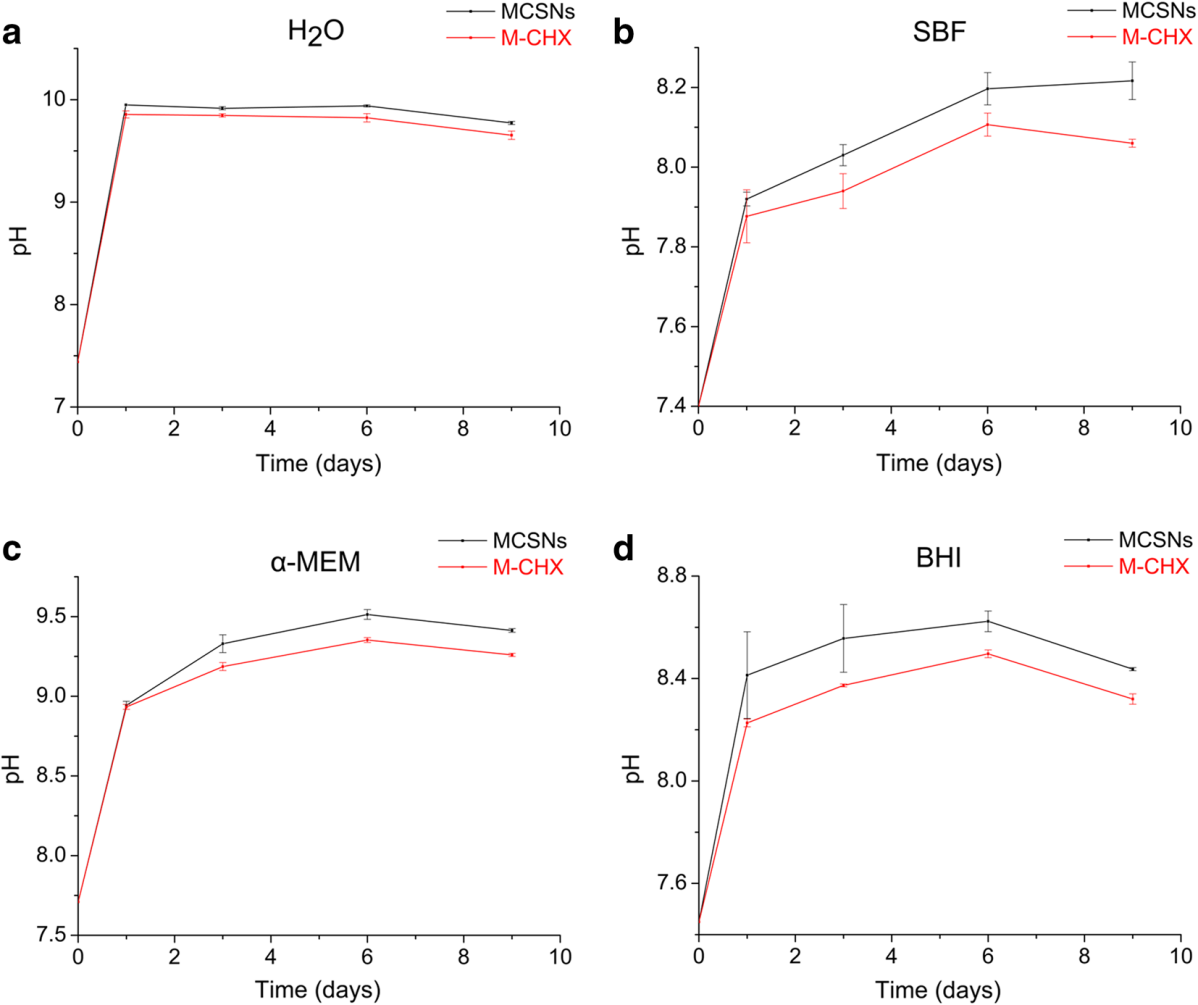
The pH measurement of MCSNs and M-CHX. **a** pH measurement in double-distilled water; **b** pH measurement in SBF; **c** pH measurement in α-MEM; **d** pH measurement in BHI

pH of both MCSNs and M-CHX were alkaline in all different solutions while pH of MCSNs was always a little higher than M-CHX (Fig. 3). In double-distilled water, MCSNs and M-CHX displayed a quite stable and highest pH (about 10) over 9 days (Fig. 3a). In SBF, pH values increased gradually to 8 and had the smallest range of variation (Fig. 3b). In α-MEM pH of MCSNs and M-CHX reached about 9.5 while 8.5 in BHI (Fig. 3c, d). pH values decreased slightly in all solutions after 6 days.

**Fig. 4:**
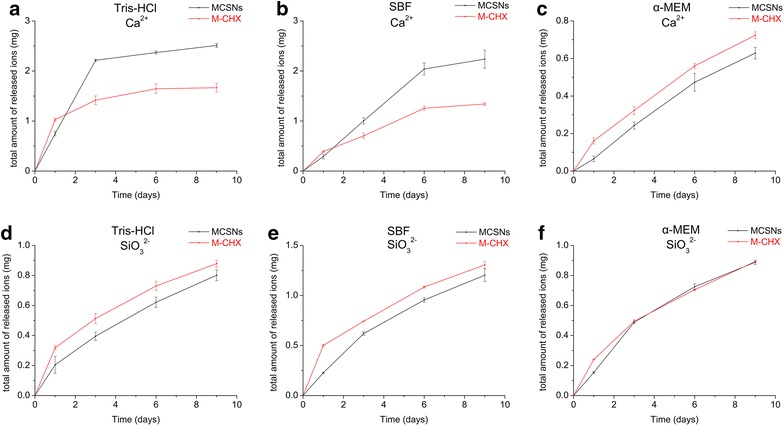
Ions release profile of MCSNs and M-CHX. **a** Ca^2+^ release profile in Tris–HCl; **b** Ca^2+^ release profile in SBF; **c** Ca^2+^ release profile in α-MEM; **d** SiO_3_^2−^ release profile in Tris–HCl; **e** SiO_3_^2−^ release profile in SBF; **f** SiO_3_^2−^ release profile in α-MEM

Both MCSNs and M-CHX could release Ca^2+^ and SiO_3_^2−^ ions in a sustained manner during a 9-day time span in different solutions (Fig. 4). The amount of released Ca^2+^ was maximum in Tris–HCl for both MCSNs and M-CHX while in α-MEM the release rate was the most stable. MCSNs could release more Ca^2+^ than M-CHX in Tris–HCl and SBF but it was reversed in α-MEM (Fig. 4a–c). For the release of SiO_3_^2−^, in SBF it reached the maximum amount. More SiO_3_^2−^ could be released from M-CHX than MCSNs in Tris–HCl and SBF while in α-MEM the amount was quite close (Fig. 4d–f).

**Fig. 5:**
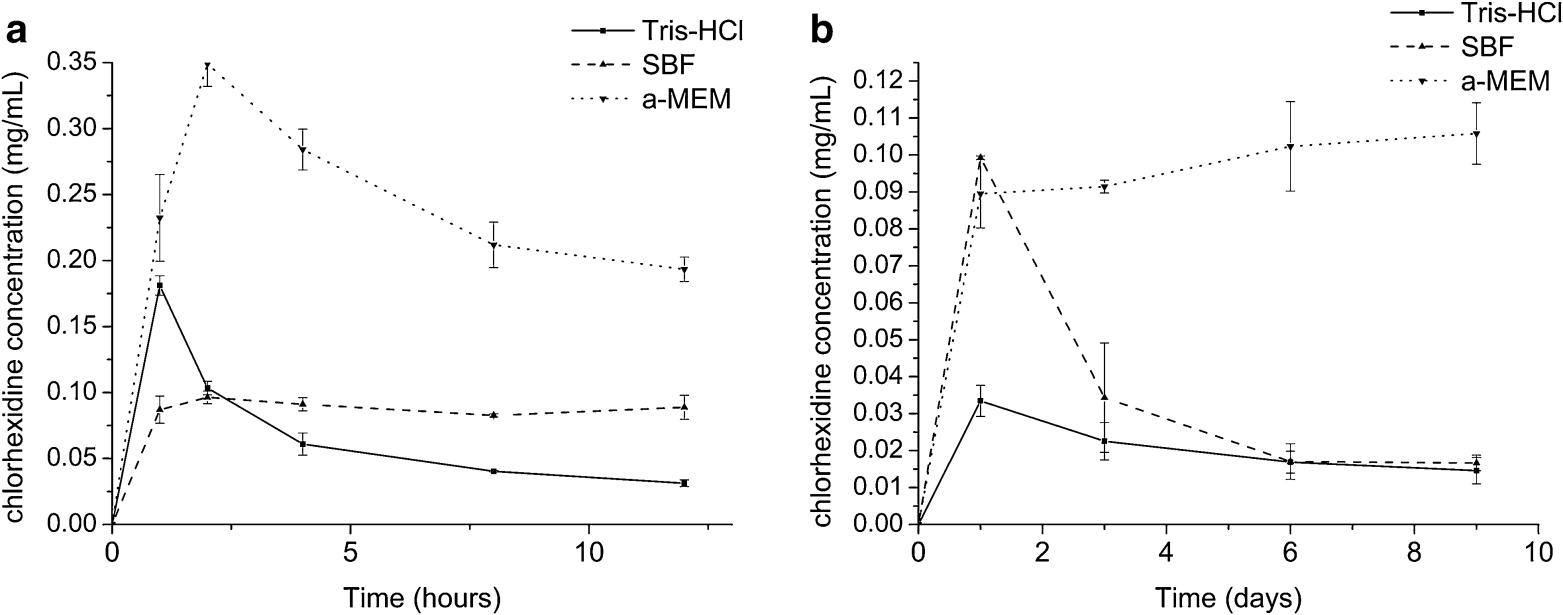
CHX release profile. **a** CHX release in 12 h; **b** CHX release in 9 days

CHX was successfully released from the M-CHX in Tris–HCl, SBF and α-MEM. In Tris–HCl and α-MEM, concentration gradually decreased after initial 2 h while in SBF the decrease began from 3 days later. In α-MEM the final concentration of CHX was the highest (Fig. 5).

### Antimicrobial effects against planktonic *E. faecalis*

**Fig. 6:**
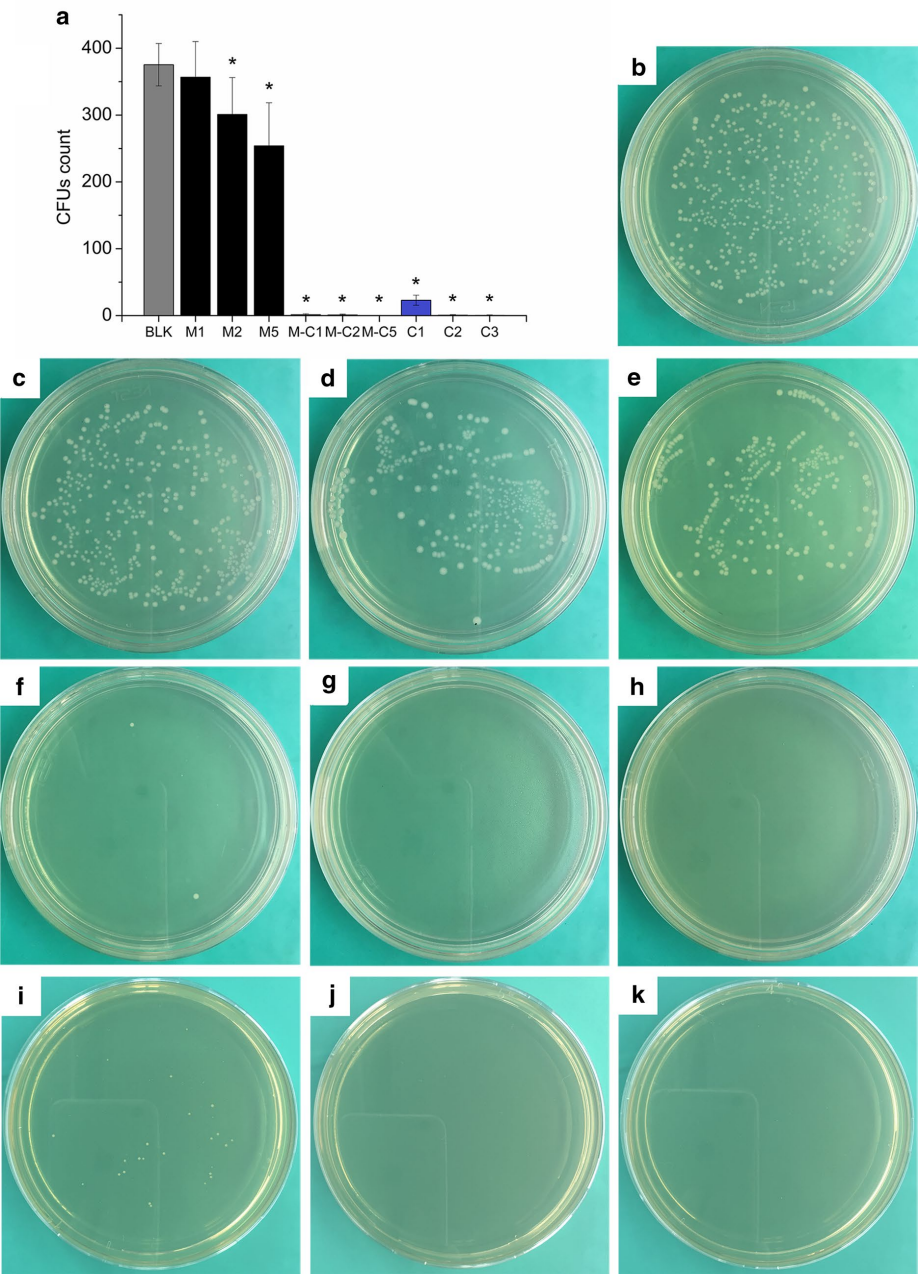
Antibacterial effects of MCSNs and M-CHX against planktonic *E. faecalis*. **a** comparisons of CFUs count among groups; **b** representative image of CFUs of negative control groups; **c–e** representative images of CFUs of 1 (**c**), 2 (**d**), 5 (**e**) mg/mL of MCSNs; **f–h** representative images of CFUs of 1 (**f**), 2 (**g**), 5 (**h**) mg/mL of M-CHX; **i–k** representative images of CFUs of 0.005 % (**i**), 0.01 % (**j**), 0.02 % (**k**) of CHX; BLK: negative control; *p < 0.05

Antibacterial test against planktonic *E. faecalis* revealed that M-CHX and CHX solution of 0.005, 0.01 and 0.02 % almost totally inhibited the growth of *E. faecalis* despite the concentrations of materials in the bacteria suspension, which was significantly better than the MCSNs and negative control groups (Fig. 6a–k; p < 0.05). The MCSNs showed a limited inhibitory effect on planktonic *E. faecalis* when the concentration of materials was more than 2 mg/mL (Fig. 6a–e; p < 0.05).

### Cytotoxicity

**Fig. 7:**
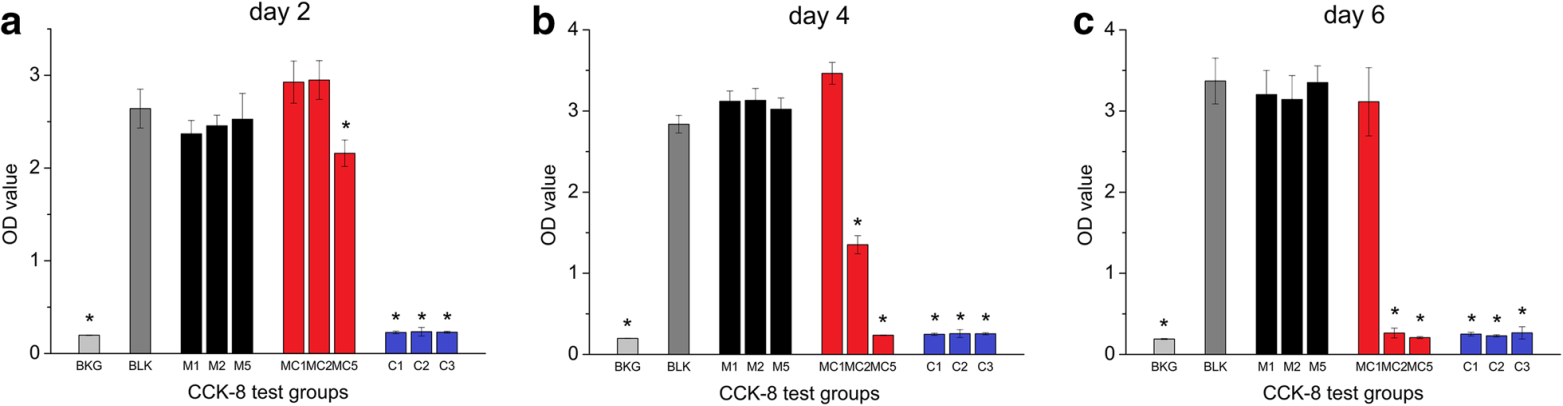
CCK-8 test results. **a** CCK-8 test result with culture time of 2 days;** b **CCK-8 test result with culture time of 4 days;** c **CCK-8 test result with culture time of 6 days. BKG: medium background; BLK: cell culture group without adding extracts; M1, 2, 5: final concentration of 1, 2, 5 mg/mL of MCSNs extracts; MC1, 2, 5: final concentration of 1, 2, 5 mg/mL of M-CHX extracts; C1, 2, 3: final concentration of 0.01, 0.02, 0.03 % of CHX; *p < 0.05

CCK-8 test on the MC3T3-E1 cells indicated that MCSNs at all three concentrations and M-CHX at a final concentration of 1 mg/mL showed no suppressive effect on the cell growth when compared with the control group despite exposed time (Fig. 7). M-CHX of a final concentration of 5 mg/mL started to show the suppressive effect on the cell growth at 2 days (Fig. 7; p < 0.05). At 4 and 6-day time points, M-CHX of 2 mg/mL also exhibited the suppressive effect while the negative effect of 5 mg/mL became more obvious (Fig. 7; p < 0.05). CHX solutions of 0.01, 0.02 and 0.03 % suppressed cell growth for all time points (Fig. 7; p < 0.05).

### MIC and MBC of MCSNs and M-CHX extracts

MIC and MBC results were shown in Table 3. MIC and MBC of MCSNs extract were undetectable for their limited antibacterial ability. MIC and MBC of M-CHX extract were both 25 mg/mL.

**Table 3:** MIC and MBC values of MCSNs and M-CHX extracts against *E. faecalis*

	MIC (mg/mL)	MBC (mg/mL)
MCSNs	–	–
M-CHX	25	25

### Inhibition of *E. faecalis* on dentin

**Fig. 8:**
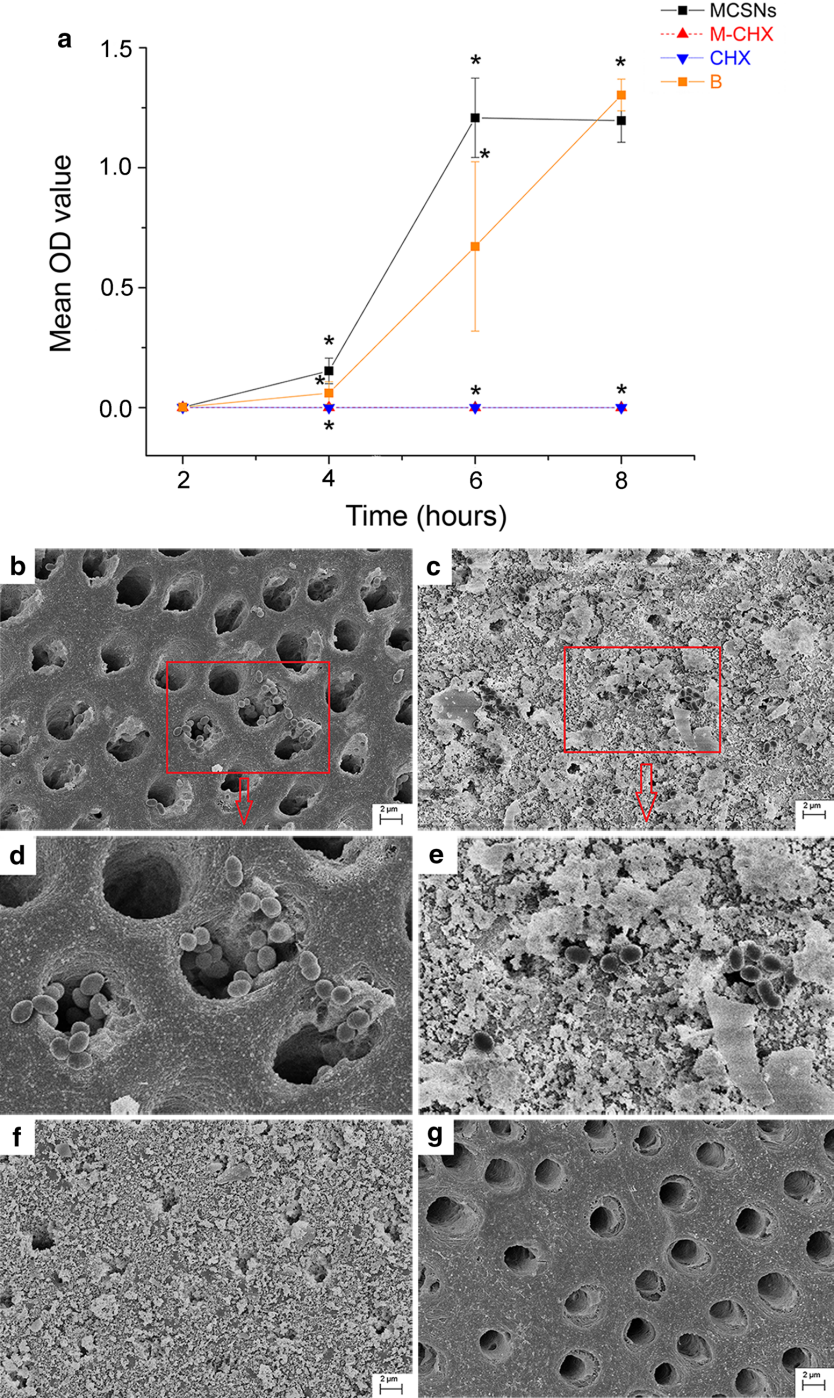
Inhibition of *E. faecalis* on dentin. **a** the curve of OD value within 8 h after the direct immerse of dentin slices incubated with *E. faecalis* for 7 days into fresh BHI solution; **b** *E. faecalis* growth on dentin slice of negative control group; **c** *E. faecalis* growth on dentin slice of MCSNs group; **d** zoom-in image from the selected area in B; **e** zoom-in image from the selected area in C; **f** *E. faecalis* growth on dentin slice of M-CHX group; **g** *E. faecalis* growth on dentin slice of CHX group; *p < 0.05

The OD value measurement revealed that almost no bacteria attached to the dentin surface medicated with M-CHX or CHX gel as the OD value did not increase during the 8 h after immersion in BHI, which was significantly different from both MCSNs and negative control groups (Fig. 8a; p < 0.05). Dentin slices without medication (negative control) or medicated with MCSNs were both infected by *E. faecalis*, and MCSNs-treated slices seemed to be more severely infected than the slices in negative control group (Fig. 8a; p < 0.05). SEM images confirmed the difference of bacteria growth on dentin surfaces of different groups. There were numerous nanoparticles left on dentin surfaces in both MCSNs group and M-CHX group even after irrigation, while original and MCSNs-treated dentin surface bonded more bacteria (Fig. 8b–g).

### In vitro mineralization

**Fig. 9:**
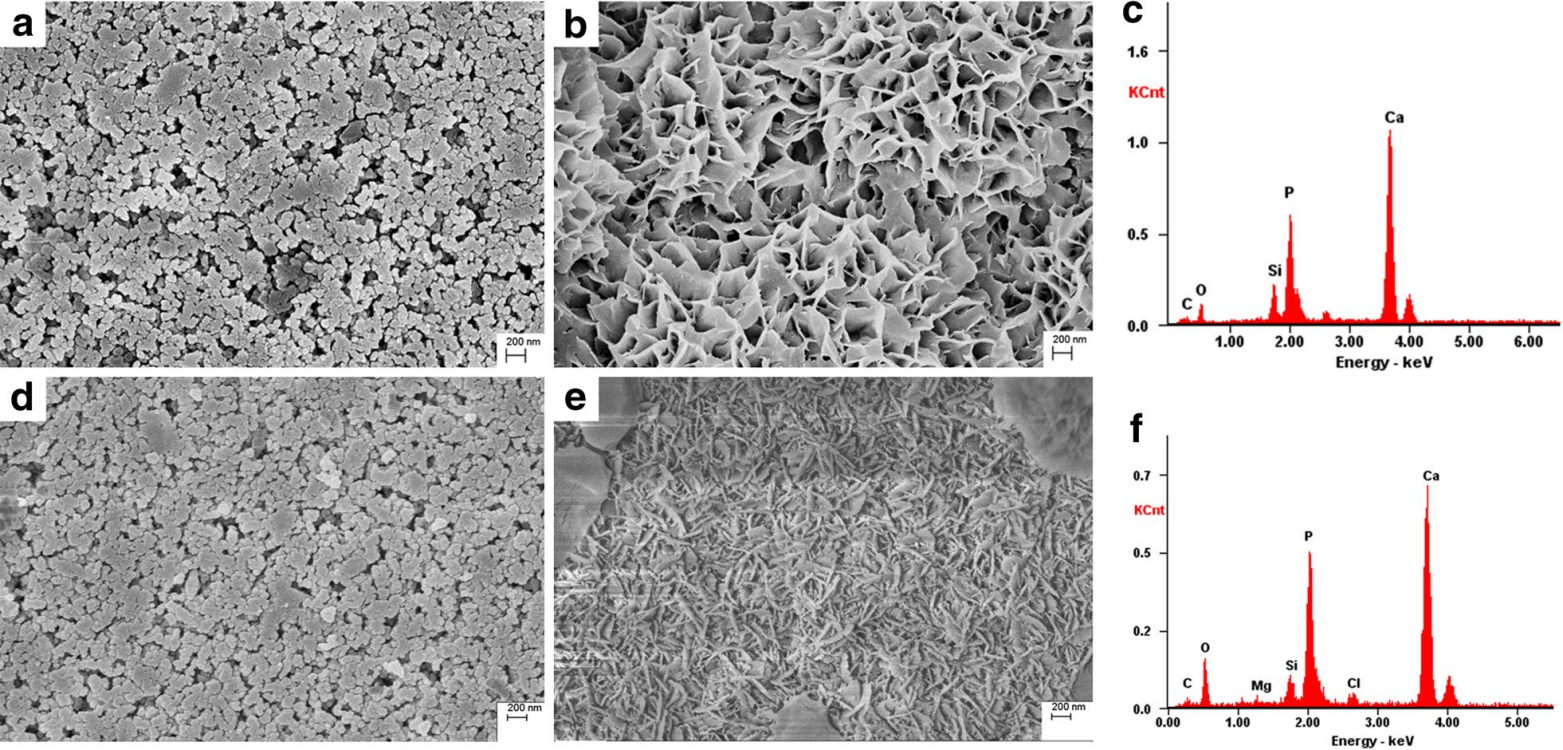
In vitro mineralization of MCSNs and M-CHX. **a** SEM image of MCSNs tablet surface; **b** SEM image of MCSNs tablet surface after being soaked in SBF for 9 days; **c** EDS spectrum of crystals formed on MCSNs tablet surface; **d** SEM image of M-CHX tablet surface; **e** SEM image of M-CHX tablet surface after being soaked in SBF for 9 days; **f** EDS spectrum of crystals formed on M-CHX tablet surface

SEM images revealed that massive apatite crystals could be induced on the surface of MCSNs and M-CHX tablets after the 9-days immersion in SBF (Fig. 9b, e). There were Ca, P, O, Si, Mg, Cl elements in these newly formed crystals according to EDS analysis (Fig. 9c, f). The Ca/P ratio was in the range of 1.5–1.8, which was consistent with the 1.67 of hydroxyapatite (HA) crystals.

## Discussion

In this study, the CHX was successfully loaded onto MCSNs through a mixing-coupling method, which was confirmed by EDS, FTIR and release spectrometry. The chemical mechanism behind this combination still needs further investigation, however, the abundant active –OH groups of MCSNs and –CH_2_ groups of CHX could very much likely bind together through covalent binding process under a heated-stirring condition. Besides, –OH groups of MCSNs and -NH- groups of CHX could also form hydrogen bonds to contribute to the combination. As the –OH groups of MCSNs are normally connected to Si^4+^, the CHX is supposed to link onto the Si^4+^ skeleton of mesoporous structures. This could partly explain the more release of SiO_3_^2−^ from M-CHX than the MCSNs together with the release of CHX in Tris–HCl and SBF. The biological significance of more SiO_3_^2−^ release especially in bone regeneration process needs to be further studied.

Though different solutions had different buffer capacity, MCSNs and M-CHX demonstrated alkaline pH in all solutions. Compared with acidic pH of CHX, this property would be more favorable for the inhibition of bacteria growth. The ions release test confirmed that M-CHX could release Ca^2+^, SiO_3_^2−^ in a sustained manner under different conditions. The media had an impact on the release of ions. A more complex media might delay ions exchange. In solutions with low ion strength, such as Tris–HCl, the release of ions would be less interfered by other ions than in solutions with high ion strength. In SBF, the release of Ca^2+^ was delayed by the relatively high concentration of Ca^2+^ in solution while the release of SiO_3_^2−^ increased as a result of the more complicated ion environment. However, with a lower Ca^2+^ concentration than in SBF, α-MEM also suppressed the release of Ca^2+^ from particles. This might be caused by the existence of organic components in α-MEM. The sustained Ca^2+^ and SiO_3_^2−^ release would favor the mineralization and crystallization, which is deemed as a very important property for new bone regeneration in vivo [30, 31]. The amount of CHX release was also different in different media. During initial several hours in Tris–HCl and α-MEM, the absorbance of solutions was relatively high. This could be caused by salts with low solubility generated by CHX and solutions. When incubation time extended, CHX concentration in media could maintain at a relatively stable level. The concentration in SBF decreased dramatically after 3 days. This phenomenon could be related to the formation of apatite crystals. In this study, the HA-like crystals were clearly observed on the surface of MCSNs and M-CHX tablets which were soaked in SBF for 9 days. It is possible that newly formed crystals absorbed part of chlorhexidine in SBF and led to the decreased concentration. But the mechanism behind this phenomenon still needs further investigation. Previous publications confirmed that bone filling materials usually form apatite on their surfaces within 4 weeks [32]. In our study, a 9-day time span was used for the observation of in vitro mineralization. On the other hand, compared with CHX, M-CHX showed very low cytotoxicity on pre-osteogenic cells at low concentration. All these features might indicate M-CHX could be developed into a new bone defect filling material.

In this study, MIC and MBC of M-CHX extracts were both 25 mg/mL. In the CHX release test, 2 mg/mL M-CHX was used. The concentration of CHX in solution was approximately in the range of 0.03–0.1 mg/mL after soaking for 24 h. When compared with other reported values, MIC and MBC values of M-CHX extracts in our study are relatively higher than MIC and MBC values of CHX [33, 34]. Except for the difference in research methods, the released amount of CHX in solution might not increase proportionally with the increasing concentration of M-CHX. However, when the nanoparticles were mixed directly with bacteria, the MIC and MBC of M-CHX particles seemed to be 5 mg/mL as shown in the antibacterial test against planktonic *E. faecalis*, which is much lower than the MIC and MBC of M-CHX extracts. The cause for this difference is not clear yet and might be the direct interact of nanoparticles with bacteria, which could possibly enhance the bactericidal effect. The antibacterial effect against planktonic *E. faecalis* of M-CHX was found very strong even at low concentrations, which is similar to CHX solutions with the matching CHX doses. As low concentration of M-CHX showed no cytotoxicity, M-CHX at low concentration would guarantee both strong antibacterial ability and bio-compatibility. 1 mg/mL seemed to be a proper concentration for M-CHX. As to the substantivity and residual inhibitory effect on the adhesion of *E. faecalis* on dentin surface, M-CHX seemed very much similar to the CHX gel, and this might indicate that the M-CHX could be a substitute for CHX gel to be used as an intra-canal medication. The mechanism for the substantivity of M-CHX on dentin surface is not clear yet, but could be that the Ca^2+^/SiO_3_^2−^ ions of M-CHX reacted and mineralized with the HA crystals on the dentin surface to form a fused mineralization layer. Besides, the negative charges on collagen fibers of dentin could attract the cationic CHX molecules and bind together as that happened between the CHX gel and dentin surface [35].

Considering the features of M-CHX found in this study, the M-CHX combined the strong anti- *E. faecalis* ability and substantivity of CHX with the pro-mineralization property of MCSNs and reduced the cytotoxicity, which could be developed into a new effective intra-canal medication in dentistry or a new bone defect filling material for infected bone defects.

## Conclusions

CHX could be successfully loaded onto the MCSNs by a mixing-coupling method. The M-CHX presented release of CHX as well as sustained release of Ca^2+^/SiO_3_^2−^ ions, low cytotoxicity, excellent anti- *E. faecalis* ability and in vitro mineralization property. These features would enable M-CHX to be developed into a new effective intra-canal medication in dentistry or a new bone defect filling material for infected bone defects.
